# Detection of occult atrial fibrillation in patients with embolic stroke of uncertain source: a work in progress

**DOI:** 10.3389/fphys.2015.00100

**Published:** 2015-04-01

**Authors:** Jason G. Andrade, Thalia Field, Paul Khairy

**Affiliations:** ^1^Electrophysiology Service, Department of Medicine, Montreal Heart Institute, Université de MontréalMontreal, QC, Canada; ^2^Department of Medicine, Division of Cardiology, University of British ColumbiaVancouver, BC, Canada; ^3^Department of Medicine, Division of Neurology, University of British ColumbiaVancouver, BC, Canada

**Keywords:** atrial fibrillation, stroke, implantable cardiac monitor, loop recorder, thromboembolism

## Abstract

Atrial fibrillation accounts for a substantial proportion of ischemic strokes of known etiology and may be responsible for an additional subset of the 25–40% of strokes of unknown cause (so-called cryptogenic). Oral anticoagulation is significantly more effective than antiplatelet therapy in the secondary prevention of atrial fibrillation-related strokes, providing justification for developing more sensitive approaches to detecting occult paroxysms of atrial fibrillation. In this article, we summarize the current state of knowledge regarding the value of in-hospital and out-patient monitoring for detecting atrial fibrillation in the context of cryptogenic stroke. We review the evidence for and against screening with standard Holter monitors, external loop recorders, the newer real-time continuous attended cardiac monitoring systems, cardiac implantable electronic devices, and insertable loop recorders. We review key questions regarding prolonged cardiac arrhythmia monitoring, including the relationship between duration of the atrial fibrillation episode and risk of thromboembolism, frequency of monitoring and its impact on the diagnostic yield in detecting occult or subclinical atrial fibrillation, and the temporal proximity of device-detected atrial fibrillation to stroke events. We conclude by proposing avenues for further research.

## Introduction

Cryptogenic stroke (CS) refers to cerebral ischemia of obscure or unknown mechanism. Specifically, it is defined as cerebral infarction that despite extensive evaluation is not attributable to a definite source of cardioembolism, large artery atherosclerosis, or small vessel disease (lacunar infarct) (Adams et al., 1993). Based on contemporary estimates, approximately 25–40% of ischemic strokes are cryptogenic (Sacco et al., 1989; Petty et al., 1999; Kolominsky-Rabas et al., 2001; Lee et al., 2001; Liao et al., 2007). The underlying mechanism of CS is often not identified because (1) the inciting mechanism for ischemia may be transitory [e.g., paroxysmal atrial fibrillation (AF)] or reversible (e.g., drug-induced vasculopathy), (2) investigations performed did not explore all possible etiologies (e.g., no blood cultures performed in the context of an infectious endocarditis), or (3) the cause remains truly unknown.

It has been postulated that additional extended electrocardiographic monitoring may identify etiologic paroxysmal AF in a subset of strokes initially categorized as cryptogenic. While AF is a common cause of ischemic stroke, representing approximately 50% of cardioembolic strokes and 20% of all strokes, underdetection remains problematic given its unreliable symptom profile (i.e., poor correlation between symptoms and AF episodes), coupled with its intermittent nature (paroxysmal AF is, by definition, a disease of episodic clusters) (Page et al., 1994; Kottkamp et al., 2004; Piorkowski et al., 2005; Strickberger et al., 2005; Ziegler et al., 2006; Cotter et al., 2013; Verma et al., 2013).

In the context of AF, oral anticoagulation reduces stroke and systemic embolic risk by two-thirds (Hart et al., 2007). Conversely, antiplatelet therapy, which remains the treatment of choice for an ischemic stroke not attributable to a major-risk cardioembolic source, is far less effective for secondary prevention in AF (Connolly et al., 2006). Consequently, if AF remains undetected, secondary prevention therapy is often suboptimal. The importance of detecting occult AF is critical considering that, compared with ischemia due to other mechanisms, AF-related strokes are associated with a higher recurrence rate over the short and long term, and greater severity. Specifically, an AF-related stroke is associated with significantly greater morbidity and long-term disability, increased in-hospital mortality, and a higher index of fatal recurrence (Bruggenjurgen et al., 2007; Winter et al., 2009). Thus, identification of appropriate candidates for anticoagulation is paramount for prevention of AF-associated thromboembolism.

## In-hospital and brief monitoring for detection of AF in patients with cryptogenic stroke

Post-stroke in-hospital rhythm monitoring is limited by a finite window of observation, which is particularly problematic in the context of intermittent AF. The detection rate of new paroxysmal AF (i.e., AF not previously identified by history or ECG) from a standard 12-lead ECG after ischemic stroke or transient ischemic attack (TIA) is estimated at 2–4% (Bell and Kapral, 2000; Jabaudon et al., 2004; Ritter et al., 2013). Conversely, continuous cardiac rhythm monitoring (CCM) for 28–72 h after index hospital admission has been reported to detect new AF in up to 2.4–18.5% of patients with acute ischemic stroke (Table 1) (Rem et al., 1985; Barthelemy et al., 2003; Sulter et al., 2003; Bansil and Karim, 2004; Adams et al., 2007; Vivanco Hidalgo et al., 2009; Rizos et al., 2010, 2012; Gumbinger et al., 2012; Kallmunzer et al., 2012; Lazzaro et al., 2012; Fujii et al., 2013; Ritter et al., 2013; Sutamnartpong et al., 2014). Despite such early intense monitoring, a substantial proportion of patients at risk for recurrent cardioembolism from occult AF remain undiagnosed.

**Table 1:** **Short-term monitoring and AF detection**.

**Sources**	**Intervention**	**Duration of monitoring**	***N***	**AF Detected (%)**
Bansil and Karim (Bansil and Karim, 2004)	CCM	NA	121	4.9
Barthelemy (Barthelemy et al., 2003)	CCM	70 h	52	7.7
Gumbinger (Gumbinger et al., 2012)	CCM	24 h	312	11.9
Kallmunzer (Kallmunzer et al., 2012)	CCM	76 h	271	6.6
Lazzaro (Lazzaro et al., 2012)	CCM	73 h	133	6.0
Rem (Rem et al., 1985)	CCM	48 h	169	2.4
Rizos (Rizos et al., 2010)	CCM	48 h	136	21.3
Rizos (Rizos et al., 2012)	CCM	64 h	496	5.4
Ritter (Ritter et al., 2013)	CCM	72 h	1110	1.3
Sulter (Sulter et al., 2003)	CCM	48 h	27	18.5
Sutamnartpong (Sutamnartpong et al., 2014)	CCM	24 h	204	5.8
Vivanco (Vivanco Hidalgo et al., 2009)	CCM	55 h	465	7.1
Fudji (Fujii et al., 2013)	CCM or Holter	NA	113	11.5
Barthelemy (Barthelemy et al., 2003)	Holter	24 h	55	5.5
Douen (Douen et al., 2008)	Holter	24 h	126	9.5
Gladstone (Gladstone et al., 2014)	Holter	24 h	277	3.2
Gumbinger (Gumbinger et al., 2012)	Holter	24 h	192	1.0
Gunalp (Gunalp et al., 2006)	Holter	24 h	26	42.3
Hornig (Hornig et al., 1996)	Holter	24 h	261	3.8
Jabaudon (Jabaudon et al., 2004)	Holter	24 h	139	5.0
Kessler (Kessler and Kessler, 1995)	Holter	24 h	93	0.0
Lazzaro (Lazzaro et al., 2012)	Holter	24 h	133	0.0
Rizos (Rizos et al., 2012)	Holter	24 h	496	2.8
Ritter (Ritter et al., 2013)	Holter	24 h	1110	0.5
Schaer (Schaer et al., 2004)	Holter	24 h	425	2.1
Shafqat (Shafqat et al., 2004)	Holter	24 h	210	2.4
Sobocinski (Doliwa Sobocinski et al., 2012)	Holter	24 h	249	2.0
Stahrenberg (Stahrenberg et al., 2010)	Holter	24 h	224	4.8
Tagawa (Tagawa et al., 2007)	Holter	24 h	299	8.4
Thakkar (Thakkar and Bagarhatta, 2014)	Holter	24 h	52	5.8
Rem (Rem et al., 1985)	Holter	24–48 h	51	3.9
Dangayach (Dangayach et al., 2011)	Holter	48 h	51	23.6
Stahrenberg (Stahrenberg et al., 2010)	Holter	48 h	224	6.4
Schuchert (Schuchert et al., 1999)	Holter	72 h	82	6.1
Stahrenberg (Stahrenberg et al., 2010)	Holter	7 d	224	12.5
Ritter (Ritter et al., 2013)	Holter	7 d	60	1.7

*MCOT, mobile cardiac outpatient telemetry; ICM, implantable cardiac monitor; ELR, external loop recorder; NA, not available*.

In patients with cryptogenic stroke in whom AF is suspected, an increased intensity of arrhythmia monitoring is generally recommended. Unfortunately, the optimal timing (e.g., from the index stroke), the method, and duration of monitoring to maximize detection of occult AF remain unclear. Traditionally, 24-h ambulatory ECG (Holter) monitoring has been employed, though the utility of Holter monitoring is limited by low rates of arrhythmia detection (~4%, Table 2), inadequate negative predictive value, and poor cost-effectiveness in unselected patients (Rem et al., 1985; Kessler and Kessler, 1995; Hornig et al., 1996; Schuchert et al., 1999; Barthelemy et al., 2003; Jabaudon et al., 2004; Schaer et al., 2004; Shafqat et al., 2004; Gunalp et al., 2006; Tagawa et al., 2007; Douen et al., 2008; Stahrenberg et al., 2010; Dangayach et al., 2011; Doliwa Sobocinski et al., 2012; Gumbinger et al., 2012; Lazzaro et al., 2012; Rizos et al., 2012; Ritter et al., 2013; Gladstone et al., 2014; Thakkar and Bagarhatta, 2014). As a result, clinical risk scores to help identify stroke patients at risk for paroxysmal AF were developed (Table 3) (Suissa et al., 2009; Fujii et al., 2013). Likewise, Wallmann et al. (2007) described findings on 24-h Holter monitoring (i.e., >70 premature atrial beats per 24 h) that predicted improved detection of AF (26%) when monitoring was extended to 7 days. Offering credence to the concept of pre-selecting patients for monitoring, a recent meta-analysis of 32 studies (observational or randomized studies of patients with ischemic stroke who underwent any cardiac monitoring for a minimum of 12 h) reported significantly greater detection rates of occult AF in selected (13.4%; 95% CI 9.0–18.4%) when compared to unselected (6.2%; 95% CI 4.4–8.3%) subjects (Kishore et al., 2014).

**Table 2:** **Medium and long-term monitoring and AF detection**.

**Sources**	***N***	**AF Definition**	**Intervention**	**Duration of monitoring**	**AF detected (%)**
Barthelemy (Barthelemy et al., 2003)	28	30 s	ELR	70 h	14.3
Jabaudon (Jabaudon et al., 2004)	88	“seconds”	ELR	7 d	5.7
Wallman (Wallmann et al., 2007)	127	30 s	ELR	7 d	14.2
Elijovich (Elijovich et al., 2009)	20	30 s	ELR	30 d	20.0
Flint (Flint et al., 2012)	236	30 s	ELR	30 d	7.0
Gaillard (Gaillard et al., 2010)	98	30 s	TTM	30 d	9
Gladstone (Gladstone et al., 2014)	280	30 s	ELR	30 d	16.1
		2.5 min			9.9
Bhatt (Bhatt et al., 2011)	62	30 s	MCOT	28 d	24
		5 min			9
Kamel (Kamel et al., 2013)	20	30 s	MCOT	21 d	0.0
Miller (Miller et al., 2013)	156	30 s	MCOT	30 d	5
Tayal (Tayal et al., 2008)	56	30 s	MCOT	21 d	5
Christensen (Christensen et al., 2014)	85	2 min	ICM	19 m	16.1
Cotter (Cotter et al., 2013)	51	2 min	ICM	7.6 m	25.5
Dion (Dion et al., 2010)	24	30 s	ICM	14.5 m	0.0
Etgen (Etgen et al., 2013)	22	6 min	ICM	12 m	27.3
Ritter (Ritter et al., 2013)	60	2 min	ICM	12.8 m	16.7
Rojo-Martinez (Rojo-Martinez et al., 2013)	101	2 min	ICM	9.4 m	33.7
Sanna (Sanna et al., 2014)	221	2 min	ICM	6 m	8.9

*MCOT, mobile cardiac outpatient telemetry; ICM, implantable cardiac monitor; ELR, external loop recorder*.

**Table 3:** **Clinical risk score**.

**STAF Score**	**Points**
**Suissa et al. (2009)**	
Age >62 years	2
NIHSS Score ≥8	1
Left atrial dilatation	2
Absence of vascular etiology^*^	3
Interpretation	
Score ≥5 = sensitivity 89%, specificity 88%	
**Fujii et al. (2013)**	
Mitral valve disease	1
NIHSS Score ≥8	1
Left atrial dilatation (>3.8 cm)	1
BNP ≥ 144 pg/ml	2
Interpretation	
Score ≥3 = sensitivity 78%, specificity 83%	

* *Defined by the absence of symptomatic extra- or intracranial stenosis ≥50%, symptomatic arterial dissection, clinico-radiological lacunar syndrome. BNP, B-type Natriuretic peptide; NIHSS, National Institutes of Health Stroke Scale; STAF, Score for the Targeting of Atrial Fibrillation*.

## Outpatient event monitoring for detection of AF in patients with cryptogenic stroke

Given that arrhythmia detection is related to total AF burden and improves with increasing intensity of monitoring, various strategies of prolonged monitoring have been employed. Event loop recorders (ELRs) are external devices that allow up to 30 days of cardiac rhythm recording. Several studies have evaluated their utility in patients with cryptogenic stroke despite standard diagnostic procedures including telemetry and/or Holter monitoring. Barthelemy et al. (2003) detected paroxysmal AF in 14.3% of 28 patients by ELR monitoring (24–162 h). Jabaudon et al. (2004) and Wallmann et al. (2007) employed 7-day ambulatory ECG monitoring in 149 and 127, patients respectively, detecting occult AF in 5.7 and 14.2%, respectively. In the latter study, AF was detected in 26% of patients with frequent APBs (>70/24 h) but only in 6.5% when APBs were infrequent. Two further observational studies examined the incremental role of prolonged (30-day) ELR monitoring after standard diagnostic procedures, documenting a new diagnosis of occult AF in 7–20% of patients (Elijovich et al., 2009; Flint et al., 2012).

In response to these observational studies, the open-label, multi-center, randomized controlled “30-Day Cardiac Event Monitor Belt for Recording Atrial Fibrillation after a Cerebral Ischemic Event (EMBRACE)” trial enrolled 572 subjects with no history of AF and cryptogenic stroke or TIA of undetermined cause within the previous 6 months (Gladstone et al., 2014). Of note, transesophageal echocardiography or intracranial vascular imaging was not required as part of the stroke workup. Patients were randomly assigned to non-invasive ambulatory ECG monitoring with a 30-day event-triggered loop recorder (ELR group) vs. conventional 24-h Holter monitoring (control). At 30 days, AF lasting 30 s or longer was detected in 16.1% in the ELR-group, as compared with 3.2% in the control group (*P* < 0.001; number needed to screen of 8). Episodes of AF lasting ≥2.5 min were noted in 9.9% in the ELR-group, as compared with 2.5% in the control group (*P* < 0.001). By 90 days, oral anticoagulant therapy had been prescribed for more individuals in the ELR-group than in the control group (18.6 vs. 11.1%; *P* = 0.01), presumably because of the higher rates of AF detection. Building on the observational evidence, the EMBRACE study demonstrated that the 30-day event-triggered recorder was significantly more effective than conventional 24-h Holter monitoring for identification of AF in patients with recent cryptogenic stroke.

## Outpatient telemetry for detection of AF in patients with cryptogenic stroke

Real-time continuous attended cardiac monitoring systems (e.g., Mobile Cardiac Outpatient Telemetry or MCOT) represent a novel form of monitoring that is designed to address the limitations of standard Holter and ELR monitoring. Specifically, while they record automatic and patient triggered events similar to an ELR, the information is sent to a central monitoring station for analysis and transmitted to the treating physician. Several studies have highlighted the utility of MCOT in detecting occult AF in the context of cryptogenic stroke with an unrevealing arrhythmia investigation, with an incidence of approximately 9% (ranging from 0–24%; Table 2) (Tayal et al., 2008; Bhatt et al., 2011; Kamel et al., 2013; Miller et al., 2013). In these series, the time to arrhythmia detection was protracted, suggesting improved arrhythmia detection with prolonged monitoring (>7 days). Unfortunately, compliance with prolonged MCOT monitoring is suboptimal, with approximately 80% of patients completing at least 14 days and 62% at least 21 days in the largest reported series (Miller et al., 2013).

## AF detection on cardiac implantable electronic devices (CIED)

**Figure 1:**
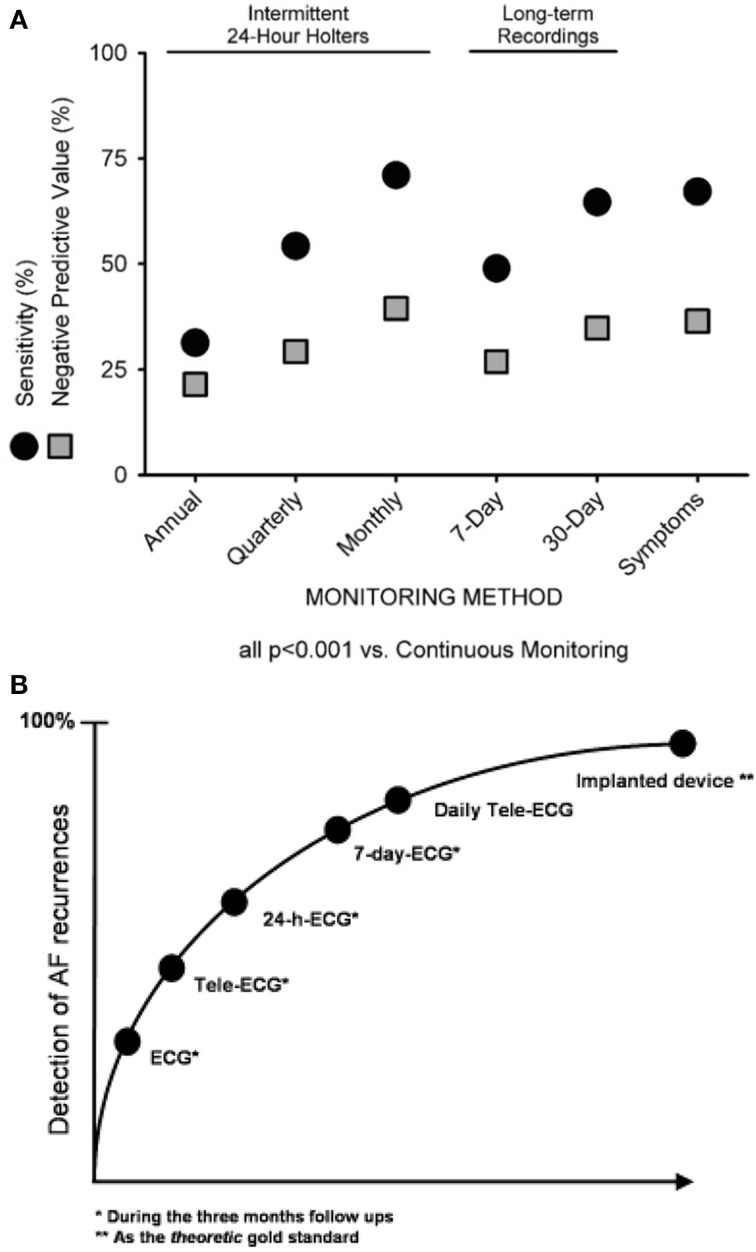
**Sensitivity and negative predictive value for identification of patients with any atrial tachycardia/atrial fibrillation (AT/AF) episodes identified by various intermittent monitoring methods, utilizing continuous monitoring as the gold standard**. Reproduced with permission from Ziegler et al. (2006) **(A)** and Arya et al. (2007) **(B)**.

Continuous ECG monitoring via CIEDs (e.g., pacemakers or defibrillators) represents the gold standard for asymptomatic arrhythmia detection, due to their ability to provide complete (uninterrupted) arrhythmia monitoring. Several studies have evaluated the performance of intermittent ECG monitoring in comparison to CIED monitoring (Figure 1). Ziegler et al. (2006) performed a retrospective analysis of 574 CIED patients who were known to have AF. In order to simulate the nature of intermittent arrhythmia monitoring, the authors assessed the detection of atrial tachyarrhythmias (AT) or AF on randomly selected days. When compared with continuous CIED monitoring, the use of intermittent techniques had a significantly lower sensitivity (31.3% for annual 24-h recordings, 54.2% for quarterly 24-h recordings, 71.0% for monthly 24-h recordings, 48.9% for 7-day monitoring, and 64.6% for 30-day monitoring) with a relatively poor negative predictive value (21.5, 29.2, 39.4, 26.9, and 34.7%, respectively). Moreover, intermittent monitoring significantly underestimated the overall AT/AF burden (*P* < 0.001). Similar findings were reported by Botto et al. (2009) in a comparable patient population whereby the sensitivity for detecting an AF episode lasting >5 min was 44.4, 50.4, and 65.1% for 24-h, 1-week, and 1-month monitoring, respectively.

Leveraging the increased sensitivity and specificity associated with these devices, there has been renewed interest in their ability to detect silent paroxysmal AF. In patients with a pacemaker or defibrillator but without a known history of AF (or of oral anticoagulation or antiarrhythmic drug use), the prevalence of previously unrecognized paroxysmal AF was 30–35% (Healey et al., 2012; Ziegler et al., 2012). In those with a history of stroke or TIA and a clinical indication for pacemaker or defibrillator implantation, interrogation of the device identified occult AF (>5-min duration) in 28% over a mean follow-up of 1.1 ± 0.7 years (Ziegler et al., 2010). In this sub-analysis of the TRENDS trial, most patients with AT/AF had infrequent episodes (73% had AT/AF on <10% of recording days), and were first detected more than 1 month after implant in 60%.

## AF detected by insertable cardiac monitors

While continuous arrhythmia monitoring provided by CIED is clearly beneficial, their use is limited to patients with a clinical indication for pacemaker or defibrillator implantation. In recent years, the focus of long-term monitoring for cryptogenic stroke has shifted toward an evaluation of dedicated subcutaneous monitors. The implantable cardiac monitor (ICM) or insertable loop recorder (ILR) is a subcutaneous device implanted in the left pectoral region that is capable of continuous uninterrupted arrhythmia monitoring. However, unlike atrial-based CIEDs, these subcutaneous monitors do not sense endocardial atrial activity well. Atrial arrhythmias are detected and classified by a dedicated AF detection algorithm. This algorithm analyzes the irregularity of successive R–R intervals over a minimum time interval (usually 2 min), classifying arrhythmias on the basis of differences between consecutive R–R segments (e.g., Lorenz scatterplots that show the R–R interval as a function of the preceding R–R intervals). The sensitivity and specificity of these monitors are limited by extraneous noise (particularly myopotentials), frequent atrial or ventricular premature beats, or pronounced sinus arrhythmia, thus necessitating visual inspection of the recorded 2-min ECG strip for confirmation. As such, while these devices are highly sensitive in detecting atrial arrhythmias (>95%), they appear to lack specificity (Hindricks et al., 2010).

Despite these potential limitations, there has been recent interest in the use of ICM-based AF detection in the evaluation of cryptogenic stroke. To date, six observational studies have assessed their dedicated AF detection algorithms as an adjunct to standard investigations including cerebral imaging, echocardiography, 12-lead ECG, inpatient telemetry, and 24-h ambulatory ECG monitoring. While Dion et al. (2010) failed to identify a single case of subclinical AF in 24 patients after a mean 14.5 months of ICM monitoring, a significant burden of subclinical AF was detected in five other observational series (despite variable screening prior to device implantation and variable duration of monitoring). The reason for this discrepancy likely lies in the former's use of an older ICM that relied on ventricular rate for detecting AF, a method that has poor sensitivity and underestimates AF burden. When devices with validated algorithms (i.e., Lorenz plots) for AF detection were utilized, a high prevalence of occult AF was detected. Christensen et al. identified subclinical AF (≥2 min) in 14 of 85 (16.1%) patients implanted with an ICM during an 18-month study period (Christensen et al., 2014). The mean time to AF detection was 109 days, and the majority of episodes were short (2–10 min duration). Cotter et al. (2013) identified subclinical AF (≥2 min) in 13 of 51 (25.5%) patients, with a median time to AF detection of 48 days. Etgen et al. (2013) used a more stringent definition (≥6 min) and identified subclinical AF in 6 of 22 (27.3%) individuals, with a median time to detection of 161 days. Rojo-Martinez et al. (2013) detected subclinical AF (≥2 min) in 34 of 101 (33.7%) individuals over a median follow-up of 281 days. Lastly, Ritter et al. (2013) evaluated 60 patients presenting with acute cryptogenic stroke. After a standard work-up, patients underwent ICM implantation an average of 13 days after presentation followed by 7-day Holter monitoring. After a minimum 12 months of follow-up, intermittent AF lasting >2 min was detected in 10 patients via ICM (17%; average time to detection of 64 days), in contrast to only 1 (1.7%) with AF detected by 7-day Holter monitoring.

Although there is a natural inclination to compare diagnostic yields between studies, it is important to consider that several factors obscure direct comparisons. Specifically, the prevalence of AF may be influenced by inherent differences in patient populations and the rigor of investigation in defining the etiology of stroke as cryptogenic (e.g., inclusion of prolonged in-hospital arrhythmia monitoring, transesophageal echocardiography (TEE), and screening for hypercoagulable states). Likewise, differences in study design could impact the amount of AF detected through the use of variable monitoring durations (i.e., sensitivity increases with monitoring time) and non-uniform definitions (e.g., higher prevalence expected with less stringent definitions of continuous AF).

In order to address some of these limitations, the Cryptogenic Stroke and underlying Atrial Fibrillation (CRYSTAL-AF) study was designed as a large, prospective, multicenter, international, randomized controlled trial (Sanna et al., 2014). A total of 441 patients with recent cryptogenic stroke or TIA (within 90 days; mean 38.1 ± 27.6 days) but without a history of AF were randomized 1:1 to standard arrhythmia monitoring (control arm; *n* = 220) vs. implantation of a subcutaneous cardiac monitor (ICM; *n* = 221). Prior to enrolment, alternate sources of stroke were excluded by 12-lead ECG, 24-h ECG monitoring, TEE, computed tomographic angiography or magnetic resonance angiography of the head and neck to rule out an arterial source, and screening for hypercoagulable states in patients younger than 55 years. The primary endpoint was time to detection of AF (lasting more than 30 s) within 6 months after stroke. The rate of AF detection at 6 months was 8.9% (*n* = 19) in the ICM group compared to 1.4% (*n* = 3) in the control group [hazard ratio (HR), 6.4; 95%CI 1.9–21.7; *P* < 0.001]. When monitoring continued from 6 through 12 months, an additional 10 first episodes of AF were detected (12.4%; *n* = 29) in the ICM group vs. 1 in the control group (2.0%; *n* = 4). The median time from randomization to detection of AF was 84 days in the ICM group and 53 days in the control group. At 12 months, 121 ECGs, 32 24-h Holter monitors, and 1 event recorder were required to identify AF in 4 patients in the standard monitoring group. The first episode of AF was asymptomatic in 23 of 29 patients randomized to ICM monitoring (79%) and in 2 of 4 patients in the control group (50%), reinforcing the limitations of symptom-driven or intermittent short-term monitoring. At 12 months, ischemic stroke or TIA occurred in 15 subjects (7.1%) in the ICM group vs. 19 (9.1%) in the control group. The most common adverse events associated with ICM were infection [3 subjects (1.4%)], pain [3 subjects (1.4%)], and irritation or inflammation [4 subjects (1.9%)] at the insertion site. The ICM remained inserted in 98.1% of subjects at 6 months and in 96.6% of subjects at 12 months.

## AF episode duration and the risk of thromboembolism

The detection of AF on prolonged cardiac monitoring is limited by the specificity of its clinical significance. As outlined above, increasing the duration and frequency of monitoring will increase the detection rate of occult or subclinical AF. However, even in the context of prior thromboembolism, the relevance of short, asymptomatic occult AF episodes remains poorly understood. To this end, several series examining occult AF on implantable devices have attempted to identify a threshold AF duration associated with adverse clinical consequences such as thromboembolism. Despite widespread interest, reported threshold durations are highly variable, ranging from 5 min by Glotzer et al. (2003, 2009) (2.8 greater risk for stroke or death), to 6 min by Healey et al. (2012) (2.5 greater risk for thromboembolism), to 24 h by Capucci et al. (2005) (3.1 greater risk for thromboembolism). Likewise, a daily burden of 3.8 h (Shanmugam et al., 2012) and 5.5 h (Glotzer et al., 2009) has also been associated with significant increases in the risk of stroke (9 and 2-fold increases, respectively).

Several authors have attempted to integrate clinical parameters with AF episode duration/burden in order to refine stroke risk stratification (Botto et al., 2009; Boriani et al., 2011). Botto et al. (2009) demonstrated that by combining AF presence/duration with the CHADS_2_ score, risk prediction could be improved: a low risk of stroke (0.8%) was observed in patients with <5 min of AF and a CHADS_2_ score ≤2, AF lasting 5 min to 24 h with and a CHADS_2_ score ≤1, and AF lasting >24 h with a CHADS_2_ score of 0. In a separate series, improved specificity was observed through an integration of the CHA_2_DS_2_VASC score and AF episode duration/burden (Boriani et al., 2011). Of note, in this population of patients with a history of paroxysmal atria tachycardias who were implanted with a dual chamber pacemaker the use of the CHA_2_DS_2_VASC score alone conferred 100% sensitivity to predict thromboembolism, albeit with poor specificity (7% for a score ≥1 and 24% for a score ≥2). However, the integration of AF presence/duration/burden improved the c-statistic from 0.653 to 0.713 for CHADS_2_ and 0.898 to 0.910 for CHA_2_DS_2_VASC (Boriani et al., 2011).

## Temporal proximity of device-detected AF to stroke events

**Figure 2:**
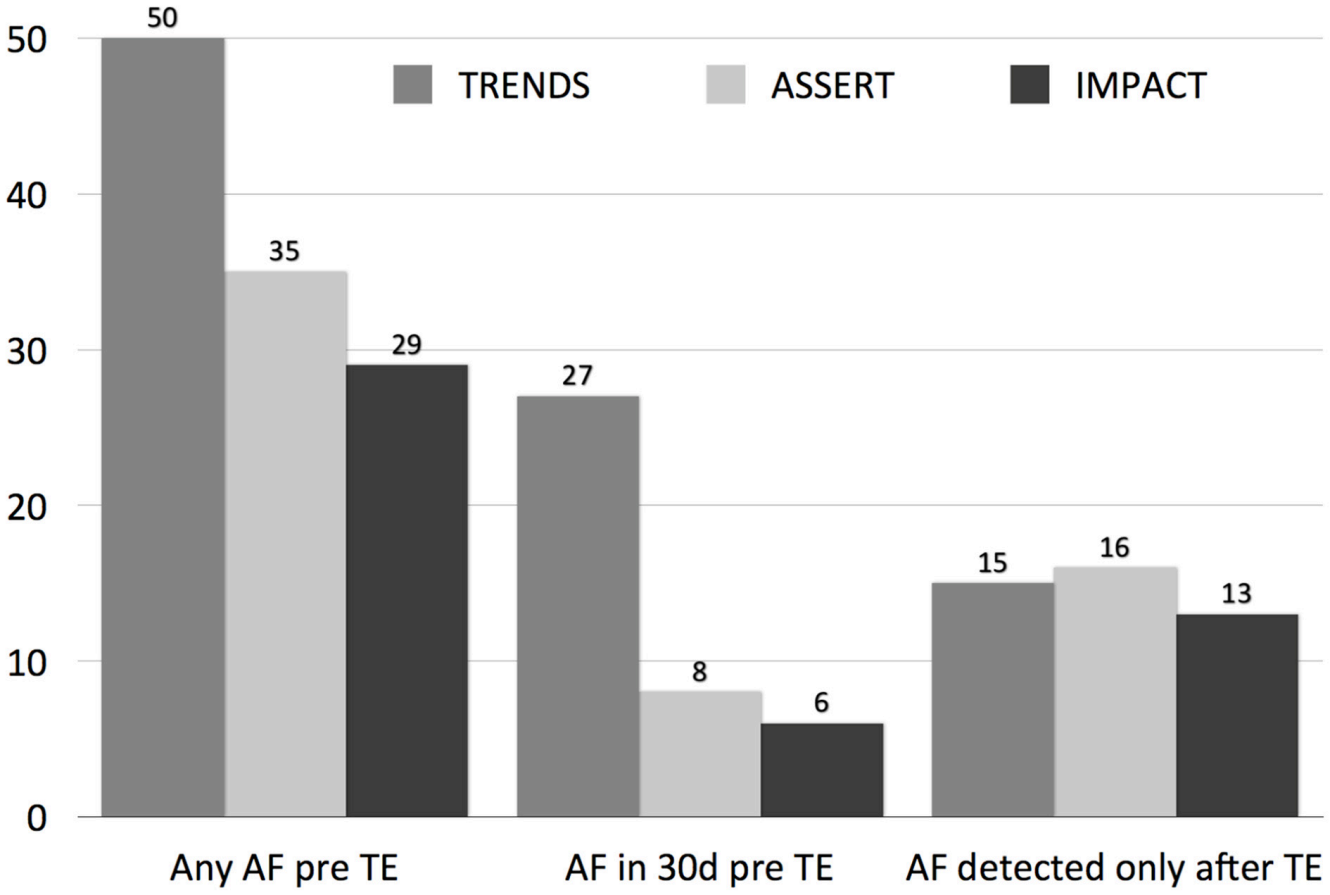
**The relationship of device-detected AF episodes to clinical thromboembolic events (TE) in three studies (ASSERT, TRENDS, and IMPACT)**. The left graph displays the prevalence of AF on CIED at any point prior to index thromboembolism (TE), the middle graph in the 30 days prior to TE, and the right graph after the index TE.

Although an association with thromboembolism has been established, short episodes of AF on long-term arrhythmia monitoring does not confer causality as the putative mechanism (Figure 2). For example, the Asymptomatic Atrial Fibrillation and Stroke Evaluation in Pacemaker Patients and the Atrial Fibrillation Reduction Atrial Pacing Trial (ASSERT) enrolled 2580 patients aged ≥65 years with hypertension but no history of atrial fibrillation. A significant association between subclinical AF (SCAF) >6 min in duration and ischemic stroke or systemic embolism was reported (hazard ratio, 2.49; 95% CI, 1.28–4.85; *P* = 0.007) (Healey et al., 2012). Interestingly, the temporal relationship between SCAF and stroke or thromboembolism was variable, with 2% of patients having SCAF at the time of stroke or systemic embolism, a further 6% having SCAF detected within 30 days before stroke or systemic embolism, 28% with SCAF detected >30 days before stroke or systemic embolism, and 16% with SCAF detected only after their stroke, despite continuous monitoring for a median duration of 228 days before their event (Brambatti et al., 2014). As such, it is possible that short episodes of AF identify patients with either more prolonged episodes of paroxysmal AF (i.e., of sufficient duration to result in thromboembolism), or alternately, act as a marker for co-morbidities that promote non-cardioembolic stroke and AF (e.g., relatively more severe hypertension).

## Conclusions and perspectives

Oral anticoagulation is substantially more effective than antiplatelet therapy for the prevention of ischemic stroke associated with AF, pr ompting much interest and progress in diagnosing occult AF in the setting of cryptogenic stroke. Technological advancements have paved the way for extended arrhythmia monitoring devices to enhance the diagnostic yield in the work-up for cryptogenic stroke by revealing brief and rare episodes of AF that would have otherwise escaped detection. Nevertheless, several uncertainties remain. Prospective studies are required to (1) evaluate the optimal duration and method of rhythm monitoring, (2) characterize the ideal population for extended rhythm monitoring, (3) determine the optimal definition of SCAF that warrants intervention (e.g., standard definition of 30 s of sustained AF vs. 6 min as used in ASSERT), and (4) evaluate whether intervention results in improved outcomes (i.e., how does initiation of oral anti-thrombotic therapy for SCAF influence the rate of stroke or systemic embolism during long-term follow-up?)

### Conflict of interest statement

The authors declare that the research was conducted in the absence of any commercial or financial relationships that could be construed as a potential conflict of interest.
